# Method: a single nucleotide polymorphism genotyping method for *Wheat streak mosaic virus*

**DOI:** 10.1186/2041-2223-3-10

**Published:** 2012-05-17

**Authors:** Stephanie M Rogers, Mark Payton, Robert W Allen, Ulrich Melcher, Jesse Carver, Jacqueline Fletcher

**Affiliations:** 1 Department of Entomology and Plant Pathology, Oklahoma State University, Stillwater, OK 74078, USA; 2 Department of Statistics, Oklahoma State University, Stillwater, OK, 74078, USA; 3 Department of Forensic Sciences, Oklahoma State University Center for Health Sciences, Tulsa, OK, 74107, USA; 4 Department of Biochemistry and Molecular Biology, Oklahoma State University, Stillwater, OK, 74078, USA

## Abstract

**Background:**

The September 11, 2001 attacks on the World Trade Center and the Pentagon increased the concern about the potential for terrorist attacks on many vulnerable sectors of the US, including agriculture. The concentrated nature of crops, easily obtainable biological agents, and highly detrimental impacts make agroterrorism a potential threat. Although procedures for an effective criminal investigation and attribution following such an attack are available, important enhancements are still needed, one of which is the capability for fine discrimination among pathogen strains. The purpose of this study was to develop a molecular typing assay for use in a forensic investigation, using *Wheat streak mosaic virus* (WSMV) as a model plant virus.

**Method:**

This genotyping technique utilizes single base primer extension to generate a genetic fingerprint. Fifteen single nucleotide polymorphisms (SNPs) within the coat protein and helper component-protease genes were selected as the genetic markers for this assay. Assay optimization and sensitivity testing was conducted using synthetic targets. WSMV strains and field isolates were collected from regions around the world and used to evaluate the assay for discrimination. The assay specificity was tested against a panel of near-neighbors consisting of genetic and environmental near-neighbors.

**Result:**

Each WSMV strain or field isolate tested produced a unique SNP fingerprint, with the exception of three isolates collected within the same geographic location that produced indistinguishable fingerprints. The results were consistent among replicates, demonstrating the reproducibility of the assay. No SNP fingerprints were generated from organisms included in the near-neighbor panel, suggesting the assay is specific for WSMV. Using synthetic targets, a complete profile could be generated from as low as 7.15 fmoles of cDNA.

**Conclusion:**

The molecular typing method presented is one tool that could be incorporated into the forensic science tool box after a thorough validation study. This method incorporates molecular biology techniques that are already well established in research and diagnostic laboratories, allowing for an easy introduction of this method into existing laboratories. Keywords: single nucleotide polymorphisms, genotyping, plant pathology, viruses, microbial forensics, Single base primer extension, SNaPshot Multiplex Kit

## Background

The September 11, 2001 attacks on the World Trade Center and the Pentagon increased concern about terrorist attacks on many vulnerable sectors of the United States (US), including agriculture [1]. The planting density of crops and the facts that biological agents are easily obtainable and can have highly detrimental impacts make agroterrorism a potential threat.

A developing area of forensic science is microbial forensics, ‘a scientific discipline dedicated to analyzing evidence from a bioterrorism act, biocrime, or inadvertent microorganism/toxin release for attribution purposes’ [2]. As the threat of the use of microorganisms as weapons has become more apparent, there is a national effort to increase capability in this new field [2,3]. Enhancements of the investigative process are still being developed to close gaps that remain in key areas, such as sample collection, packaging, shipping and storage, as well as pathogen identification and discrimination [2,4].

In addition to identifying the perpetrator of a biological crime, it is also important to identify the source of the pathogen and when and how it was introduced [4]. The stringent demands of a criminal investigation and the pressures of the courtroom require that existing methods of plant pathogen detection and disease diagnosis [5,6] be revised and validated for forensic use [4].

Molecular comparisons using single nucleotide polymorphisms (SNPs) are often used for evolutionary, ecological and forensic studies [7-15], and can be especially helpful in typing organisms having high mutation rates, such as RNA viruses, in which mutations are caused primarily by an error-prone RNA-dependent RNA polymerase [16]. Specific regions within the genome can be targeted based on the level of conservation of the nucleic acid sequence. For example, the coat protein (CP) gene of *Wheat streak mosaic virus* (WSMV) is the most variable region of the genome, with several conserved regions corresponding to the functionally important residues [17]. Other genes with highly variable regions include helper-component protease (HC-Pro) protein 1 (P1), and protein 3 (P3) [17,18]. The high mutation rate characteristic of RNA viruses contributes to the evolution of this virus and to the presence of multiple genetic variants in a single population. Analyzing the SNPs of these variants can facilitate discrimination among populations, making this procedure a powerful tool that provides benefits that techniques such as sequencing, which focus on identifying the consensus of the population, do not offer.

WSMV, an important pathogen of wheat in the US, was selected as the model pathogen for the development of a molecular typing method. WSMV belongs to the genus *Tritimovirus* within the family *Potyviridae*[19]. The 9,384 nucleotide (nt), positive sense, single-stranded RNA virus genome [20] is translated into a single polyprotein that is then processed by three viral proteinases [19]. Because most plant viruses possess an RNA genome, the method described here can be applied almost universally, with only minor modifications, to most plant viruses. Wheat streak mosaic (WSM), the disease caused by WSMV, is found in most of the wheat growing regions of the US nearly every year. Therefore, the high prevalence of WSMV facilitates collection of field isolates from across the nation, as well as internationally. Previous studies have focused on WSMV’s genetic diversity and evolution, including mutation rates, phylogenetic positions and polymorphic sites [16,18,19,21-24]. Excellent PCR detection methods have been developed and provide a basis for generating the cDNA that is subjected to the SNP typing method described here [25-27]. Extensive knowledge of the viral genome and the availability of robust detection assays facilitate the development of a more stringent, discriminatory assay for variant characterization.

Even though viruses do not represent the majority of plant pathogens, the small size and simplicity of their genomes make them great laboratory models. Bacteria and fungi, the most predominant plant pathogens, present their own unique challenges for genetic analysis, such as genome size, multiple life stages, diploid genomes, and presence of plasmids [28]. However, a method like the one presented here could be adapted for such organisms by applying what is known about the organism’s lifecycle, genomic features, and mutation rates to the selection of genetic markers and reaction conditions.

In this work, a SNP method for molecular typing of WSMV was developed by adapting protocols designed originally to test human DNA in forensic applications [7,9,12,13,29]. Fifteen SNPs, located in the CP and HC-Pro genes, were included to generate a genetic fingerprint using the ABI PRISM® SNaPshot™ Multiplex Kit. This procedure provides a mechanism of SNP identification by utilizing only fluorescently labeled dideoxynucleotides (ddNTPs) during the elongation of template-specific primers during thermocycling. The chemical composition of ddNTPs prevents any further elongation, resulting in a product that is one nucleotide longer than the primer itself. This nucleotide represents the SNP element and can be detected by fluorescence scanning of a capillary electrophoretic separation of products. We evaluated the method by determining the assay sensitivity, specificity and reproducibility, as well as its ability to discriminate among known strains and several field isolates of WSMV.

## Results

### Generation of SNP-specific primers

One hundred and twenty-two SNPs within the CP of WSMV were previously identified [19]. Ten of these SNPs were selected for inclusion in this assay because of their high variability among strains and the presence of a semi-conserved region adjacent to the SNP, providing a suitable template for primer binding. The remaining CP SNPs for this assay were identified by multiple sequence alignment of 85 different WSMV sequences obtained from the National Center for Biotechnology Information (NCBI), including the strains Sidney 81, Type, and El Batan 3 using the same criteria. The SNPs in the HC-Pro region of the genome were identified by performing a multiple sequence alignment of 13 WSMV HC-Pro sequences obtained from the NCBI database. The same criteria were used for selection of the SNPs. Due to the limited number of SNPs having an adjacent template sufficient for primer binding, both the positive and negative sense strands of the viral template were used, an approach previously used [30,31].

A 20 to 25 nt hybridizing region was designed for each SNP with the addition of a [GACT]_n_ tail to the 5′-end to provide a unique length for each primer, ranging from 30 to 65 nt. Using Oligocalc software [32], the thermodynamics and folding structures were checked for each primer. Each primer was checked for specificity to WSMV using the Basic Local Alignment Search Tool for nucleotides (BLASTn) with the available NCBI database. Any primer that was not specific to WSMV or possessed folding structures with a ∆G greater than 3.0 kcal/mol was eliminated from the study.

**Figure 1:**
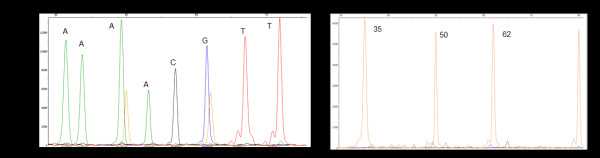
**WSMV CP multiplex electropherogram.** The height of each peak indicates the concentration of fluorescent units and the location of the peaks along the x-axis indicates the size of the fragment in nucleotides; left to right showing smallest to largest. A) Amplification of synthetic targets using eight SNP-specific primers for the WSMV CP, showing a single amplified target for each primer. B) Negative control (using sterile nanopure water instead of the synthetic templates) for the CP amplification demonstrating the absence of non-target amplification. The peaks present (labeled according to fragment size) are size standards to help with the assignment of the fragment sizes. CP, coat protein; SNP, single nucleotide polymorphism; WSMV, *Wheat streak mosaic virus*.

The remaining SNP-specific primers were screened for inclusion in the assay by performing monoplex and multiplex *in vitro* tests using synthetic targets that were specific to each primer. Primers generating a single fluorescent peak in the SNaPshot electropherogram and no peaks in the negative control were selected for inclusion in the WSMV SNaPshot assay (Figure 1). These final fifteen primers are listed in Table 1.

**Table 1:** SNaPshot primers designed for WSMV

**Name**^**a**^	**Length (nt)**	**Sequence (5′-3′)**^**b**^	**T**_**M**_	**Strand**^**c**^	**Final Conc. (μM)**
30 C	30	GACTGGCGTGTTCTCCCTCACATCATCTGC	65.1	F	0.1
35 C	35	GACTGACTGACAACTGAACAACTCAACACCTGGAT	63.5	R	0.2
40 C	40	GACTGACTGACTGACTGGCATATCTGTTGTCGATAAGTTC	63.5	F	0.1
45 C	45	GACTGACTGACTGACTGACTGTAGTTTCTACTGTGCTCACGCAAG	66.5	F	0.2
50 C	50	GACTGACTGACTGACTGACTGACTTTCGCAGCTTTGTACATCGGTTCAAT	67.6	F	0.4
55 C	55	GACTGACTGACTGACTGACTGACTGACTGATCTCTGGGCACGTTGTGTTGATTAT	72.05	F	0.4
60 C	60	GACTGACTGACTGACTGACTGACTGACTGACTGACCCCTTGGTATGATAGGTTTTCCAAT	72.82	F	0.4
65 C	65	GACTGACTGACTGACTGACTGACTGACTGACTGACTGACTTGTTCTGTATTCCGCGTAGCCTGTT	74.74	F	0.4
30 H	30	GACTGCAAGTTGTCTTAGCACATCACCAAA	60.8	F	0.4
35 H	35	GACTGACTGACTGACTGCGCAAGTTACCTGGAAGC	66.3	R	0.4
40 H	40	GACTGACTGACTGACTGCGTATCCAGACAGCGATGTAACA	66.4	R	0.4
45 H	45	GACTGACTGACTGACTGACTGAATTGTTCCATCTTCTAGCATCTT	64.7	F	0.1
50 H	50	GACTGACTGACTGACTGACTGACTGACTCGAATTTTAGGTTCGTTGATTT	65.8	R	0.1
55 H	55	GACTGACTGACTGACTGACTGACTGACTGACTTTAGACTCGCCGTTAATTTCCAT	71.31	F	0,4
60 H	60	GACTGACTGACTGACTGACTGACTGACTGACTGACTGATTTGTCACTGGGAAGGAAATGT	72.82	R	0.2

^a^The letter of the primer name indicates the target is either the coat protein (C) or the helper-component protease (H) segment of the genome.

^b^Each primer contains a segment that anneals to the template (underlined) and a GACT tail to adjust the length of the primer.

^c^The primer anneals to either the forward (F) or reverse (R) strand. nt, nucleotide; WSMV, *Wheat streak mosaic virus.*

### Sensitivity and limit of detection

**Figure 2:**
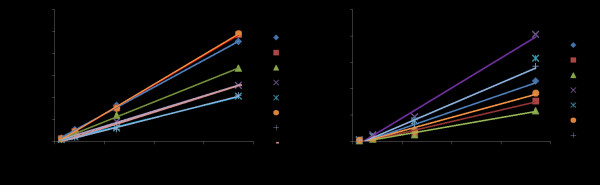
**Sensitivity of SNaPshot assay for the (A) CP and the (B) HC-Pro primers.** Both graphs demonstrate a linear relationship for each SNP (individual lines; see Figure 1 for product names). The sensitivity of the assay was determined by identifying the pmoles that correspond to 800 FU using the linear equation of the SNP with the steepest slope (C55 and H45). Linear equations: y = 336285x-475.19 and y = 285041x-1239, CP and HC-Pro, respectively. CP, coat protein; FU, fluorescent units, HC-Pro, helper-component protease, SNP, single nucleotide polymorphism.

The sensitivities of the CP and HC-Pro assays were determined using the plasmids containing the synthetic target regions. A 1:3 dilution series was created from a starting solution of 0.075 pmole/μl. The SNaPshot reaction was performed and the electropherogram generated by capillary electrophoresis. There was a linear relationship between the fluorescent units and the amount of DNA (pmoles) in each sample (Figure 2).

The background noise in the electropherograms of the negative and positive controls appeared consistently between 300 and 750 fluorescent units (FUs) (data not shown). To reduce the possibilities of false positives from background peaks, the limit of detection was set at 800 FUs, which was subsequently subtracted from all fluorescent values. The sensitivity of detection was determined by calculating the number of pmoles that correspond to 800 FUs; 3.79 fmoles and 7.15 fmoles for CP and HC-Pro, respectively.

### Specificity to WSMV

**Figure 3:**
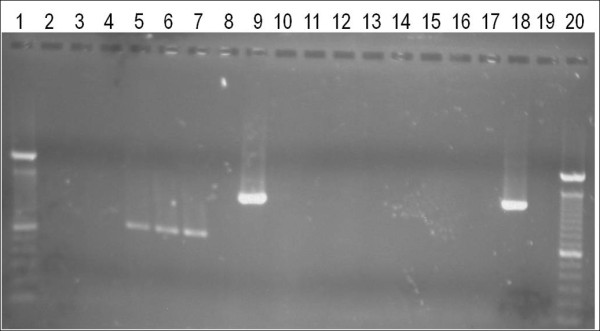
**Gel electrophoresis of CP and HCPro amplification products from WSMV near-neighbors.** Lanes 2–8 and 11–17 are ONMV, WSSMV, MDMV, HPV, CYDV-RPV, BYDV-PAV, and healthy wheat PCR products for CP and HC-Pro assays, respectively. Lanes 1 and 20 are a 100 bp DNA ladder. Lanes 9 and 18 are the positive control CP and HC-PRO WSMV plasmid, respectively. Lanes 10 and 19 are negative controls. Only the positive controls produced approximately 1,300 bp amplicons, corresponding to WSMV. BYDV-PAV, *Barley yellow dwarf virus* PAV; CP, coat protein; CYDV-RPV, *Cereal yellow dwarf virus* RPV; HC-Pro, helper-component; HPV, *High plains virus*; MDMV, *Maize dwarf mosaic virus*; ONMV, *Oat necrotic mottle virus*; PCR, polymerase chain reaction; WSMV, *Wheat streak mosaic virus*; WSSMV, *Wheat spindle streak mosaic virus*.

A panel of near-neighbors to WSMV was tested for SNaPshot assay detection using the same protocols as outlined for WSMV samples. This panel included the phylogenetic near neighbors *Oat necrotic mottle virus* (ONMV) and *Wheat spindle streak mosaic virus* (WSSMV), and the environmental near-neighbors *Maize dwarf mosaic virus* (MDMV), *High plains virus* (HPV)*, Barley yellow dwarf virus* PAV (BYDV-PAV) and *Cereal yellow dwarf virus* RPV (CYDV-RPV), as well as the healthy host *Triticum aestivum* cv. Chisholm. Gel electrophoresis results demonstrated the lack of the WSMV characteristic approximately 1,300 bp amplicon in the near-neighbor samples and the negative controls and the presence of this amplicon in the WSMV positive controls (Figure 3). Amplicons were present in three samples from the CP assay: HPV, CYDV-RPV, and BYDV-PAV. *In silico* analysis using NCBI BLASTn demonstrated that portions of the C1 and XC1 primers are complementary to regions within each of their genomes. However, the primer sequences are not 100% complementary to any of the three, which may explain the presence of the faint bands. When these three samples were subjected to the SNaPshot assay no fluorescent peaks were present in electropherograms, demonstrating that the SNaPshot assay is specific to WSMV.

### Characterization of WSMV strains and field isolates

The discriminatory capability of the SNaPshot assay was tested using known strains of WSMV (Sidney 81, Type, OSU, Merredin 1, Merredin 2, and Ginnindera) and 16 field isolates. A PCR amplicon of the appropriate size for the CP and HC-Pro genes was obtained for each sample (Figure 3). Each strain and most field isolates produced a unique SNaPshot fingerprint representing the genetic variants within each sample (Table 2). However, some field isolates collected from a common geographical region had nearly identical fingerprints, such as three samples from Montana (Conrad 1, Kalispell, and Huntley B).

**Table 2:** **SNaPshot fingerprints of WSMV strains and field isolates using the IUB nucleotide code**^**a**^

**Sample**	**Origin**	**SNP Profile**														
		**HC-Pro**							**CP**							
Sidney 81	Nebraska	M	C	C	A	G	C	G	A	W	R	R	Y	G	Y	W
Type	Kansas	K	Y	Y	A	G	M	G	A	A	C	A	T	G	C	C
OSU	Oklahoma	B	Y	Y	A	G	B	G	A	A	C	A	Y	G	Y	W
Merredin 1	Australia	K	C	C	A	G	C	G	A	W	S	R	T	G	C	W
Merredin 2	Australia	K	Y	Y	A	G	S	G	A	A	C	A	T	G	C	W
Ginnindera	Australia	K	Y	Y	A	G	V	G	A	A	C	A	T	G	T	T
Billings	Montana	T	C	C	A	G	C	G	A	A	A	A	Y	R	Y	Y
Conrad1	Montana	C	C	C	A	G	C	G	A	A	A	A	C	A	T	T
Conrad2	Montana	T	M	T	A	G	C	G	R	A	A	M	Y	R	Y	Y
Conrad3	Montana	T	C	T	A	A	C	G	R	A	C	A	T	G	C	C
Helena	Montana	C	C	C	A	G	C	G	A	A	M	A	Y	G	Y	T
HuntleyA	Montana	Y	C	C	A	G	C	G	A	A	A	A	Y	A	Y	Y
HuntleyB	Montana	C	C	C	A	G	C	G	A	A	A	A	C	A	T	T
Kalispell	Montana	C	C	C	A	G	C	G	A	A	A	A	C	A	T	T
Toole	Montana	T	C	C	A	G	C	G	A	A	-	A	T	G	C	C
OK-KF	Oklahoma	K	C	Y	A	G	S	G	A	A	S	R	C	G	Y	W
OK-CC	Oklahoma	Y	C	C	A	G	C	G	A	A	A	A	C	G	T	T
OK-Lab	Oklahoma	B	C	Y	A	G	C	G	A	A	M	A	Y	G	B	Y
OK-425	Oklahoma	K	C	C	A	G	C	G	W	A	V	R	H	S	B	W
CO-17	Colorado	K	C	C	R	G	C	G	A	A	M	A	Y	G	Y	H
NE-932	Nebraska	K	C	S	A	G	C	G	A	A	V	R	T	G	C	H
KS-117	Kansas	K	C	Y	A	G	S	G	A	A	S	R	C	G	Y	W
^b^El Batan 3	Mexico	A	C	C	A	C	T	C	A	C	G	A	C	G	C	C
^b^Czech	Czech	T	A	T	G	A	G	T	G	C	T	C	C	G	T	C

^a^IUB nucleotide code: R = AG, Y = TC, K = TG, M = AC, S = GC, W = AT, B = CGT, D = AGT, H = ACT, V = ACG, N = AGCT [33]. ^b^indicates *in silico* results based on the sequence obtained from GenBank. WSMV, *Wheat streak mosaic virus.*

**Figure 4:**
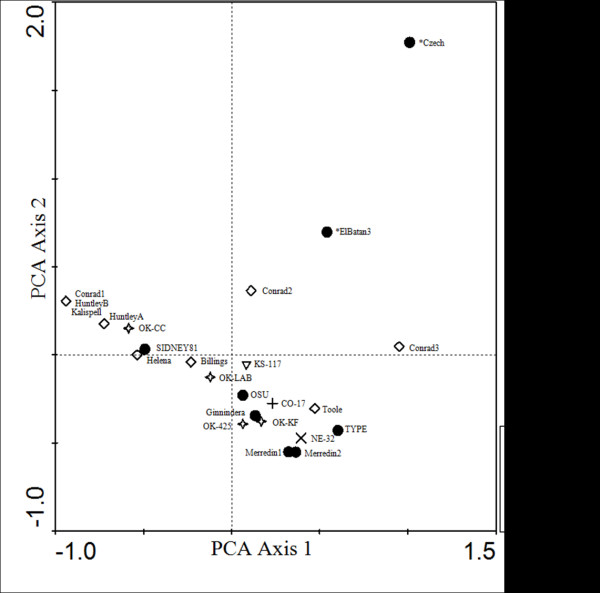
**Principle component analysis of WSMV strains and field isolates.** The majority of the samples form a weak cluster between the Sidney 81 and Type strains, with El Batan 3 and Czech strains appearing as outliers. * = profiles generated *in silico* using NCBI sequence. WSMV, *Wheat streak mosaic virus*.

The relationships among these samples were depicted using principal component analysis (PCA) with the inclusion of the *in silico* SNaPshot results of El Batan 3 and Czech WSMV strains (Figure 4). The strains Czech, El Batan 3, Sidney 81, and Type provide a dimension of diversity as these strains have been thoroughly tested in previous phylogenetic studies [16,19,23,24,34]. The genetic variability among these samples as depicted in the PCA is consistent with the current understanding of WSMV genetic diversity. The known strains and the field isolates show a high level of diversity with no apparent segregation of field isolates based on geographic location. The results indicate that these samples, even those obtained from Australia, are more closely related to Sidney 81 and Type than to El Batan 3 or Czech, a conclusion that is consistent with previous analyses of the US WSMV population [16,18,21,24].

### Reproducibility of assay

To test for reproducibility of the assay, each SNaPshot profile was generated three times from cDNA of the six WSMV strains. Each replicate produced identical SNaPshot fingerprints for each sample based on qualitative results (data not shown). To determine if the results (fluorescent units) were quantitatively reproducible, analysis of variance (ANOVA) was performed (see materials and methods for complete description of analysis). During the analysis, each nucleotide was compared to the respective nucleotide within each SNP position for each sample replicate. A subsequent analysis was performed using the individual *P*-values for each replicate group to calculate an overall *P*-value for each nucleotide. The level of variance for each nucleotide was determined based on these *P*-values. Based on the results of the peak height-absolute fluorescence dataset analysis, the fluorescence values for all four nucleotides were statistically significant (Table 3). However, only the fluorescence values of nucleotides G and T were found to be statistically significant when the analysis was performed on the peak height-nucleotide percent dataset. The same results were found for both of the area under the curve datasets with the exception of nucleotide G in the area under the curve – nucleotide percent data. The statistical significance indicates that the fluorescence values are different among each strain replicate; however, the difference is minimal when using the area under the curve dataset.

**Table 3:** ANOVA results of WSMV strain replicates

**Dataset**	**Effect**	**Nucleotide**	Num DF*	**Den DF**^+^	**F Value**	**Pr > F**^±^
Peak height – percent						
	Replicate	A	2	178	0.35	0.7075
	Replicate	C	2	178	0.64	0.5266
	Replicate	G	2	178	4.88	0.0087
	Replicate	T	2	178	3.44	0.0343
Peak height – absolute						
	Replicate	A	2	178	13.22	<0.0001
	Replicate	C	2	178	4.05	0.0190
	Replicate	G	2	178	17.73	<0.0001
	Replicate	T	2	178	11.72	<0.0001
Area under curve – percent						
	Replicate	A	2	178	0.96	0.3845
	Replicate	C	2	178	1.46	0.2342
	Replicate	G	2	178	0.75	0.4757
	Replicate	T	2	178	3.73	0.0259
Area under curve - absolute						
	Replicate	A	2	178	7.57	0.0007
	Replicate	C	2	178	10.30	<0.0001
	Replicate	G	2	178	8.21	0.0004
	Replicate	T	2	178	3.35	0.0375

*Num DF = Numerator Degrees of Freedom; +Den DF = Denominator Degrees of Freedom;.

±results are significant if Pr value is less than 0.05. WSMV, *Wheat streak mosaic virus.*

## Discussion

The ability to discriminate among viral isolates can be useful in evolutionary and ecological studies and for forensic investigations in which determining the origin of a biological agent is critical to criminal attribution. For example, identifying the source of the pathogen became a crucial aspect of the investigation of the anthrax letter attacks in 2001, but because appropriate scientific tools for strain discrimination were not available at that time it was not until seven years later that a suitable test was developed and validated, ultimately leading to attribution [35]. The development and validation of universal, robust, sensitive, and specific detection and discriminatory assays is important for a rapid and efficient response to biological attacks that may occur against our agricultural industry.

The method described here is one example of an assay that addresses this need. During a criminal investigation of a WSMV outbreak, this SNP typing method can be used to generate a genetic profile of the outbreak strain. This profile can then be compared to profiles generated from reference WSMV samples or samples found in a suspect’s possession. The results of the comparison may provide guidance to investigators in determining a likely geographic location of the origin of the virus. The unique patterns in the profile that are known to exist in a specific WSMV strain could help pinpoint the location because certain strains are known to persist in specific areas of the world. Ultimately, the strength in this assay is in comparing one sample to another to determine suspect exclusion for the crime. Determining the threshold of exclusion in terms of number of similar SNPs needs to be determined after further validation testing has occurred.

The SNP assay described here is intended for use in a forensic investigation, but it can be useful also for other molecular studies in which viral variant discrimination may be necessary. Due to the inclusion of 15 SNPs in the assay, we were able to discriminate between multiple strains and isolates of WSMV. The relationship between SNP number and discrimination ability is comparable to that of human DNA typing, in which the use of as many as thirteen short tandem repeat (STR) loci results in an extremely low probability that a genetic fingerprint could be found in any other individual based on allele frequencies in the population [36]. Because none of the probes failed to reveal a variation, each SNP is informative and should be included in the assay. Although we did not test strain El Batan 3 or any of the European strains *in vitro*, their theoretical profiles were included in the data analysis to demonstrate the estimated discriminatory ability of the method. The fifteen SNPs we used for WSMV provide a high level of confidence that the fingerprint is unique to the variant population without testing a prohibitive number of SNPs.

In our laboratory, the SNP method discriminated among several known strains of the virus and also among field isolates collected throughout the US and in Australia. Several of the profiles are similar due to most of the strains and isolates belonging to the same phylogenetic clade of WSMV [19], but minor differences exist in the secondary peaks that appear for some of the SNPs. These secondary peaks are due to the presence of multiple variants in the population. The ability for this SNP typing assay to detect multiple variants in the population is a feature that adds a higher level of discrimination than can be achieved through consensus sequence analysis. However, to fully understand if all variants or just the dominant variants are represented in the SNP profile, further testing needs to be conducted. In our hands, the limit of detection for the CP assay and HC-Pro assay, respectively, were 3.79 fmoles and 7.15 fmoles of WSMV cDNA, both of which are lower than the detection limit of 10 fmoles reported in the SNaPshot Kit manual. The level of sensitivity of this assay is comparable to that of other SNP-based techniques, reverse-transcription PCR (RT-PCR), and real-time PCR [5].

Including an internal control is important for assay validation. One of the SNaPshot primers (C55) was initially included in the assay to serve as the internal control. This primer is an excerpt of the WSMV diagnostic primer used by National Plant Diagnostic Network laboratories [37-39]. Unfortunately, this SNaPshot primer did not appear to be universal for all WSMV isolates tested. Therefore, it was no longer considered the internal control. To validate this assay for forensic investigations, an alternative internal control would need to be identified and included in the assay. At that point, a thorough evaluation of error rate would also be conducted for the assay.

In applications of the SNaPshot assay to field samples it is important to consider that many organisms and viruses other than the target may be encountered, and that some of these, such as *Triticum mosaic virus* (TriMV) and HPV, which are commonly found together in the same infected tissue [40-42], may be genetically related to WSMV. We tested many of the viruses known to co-infect wheat with WSMV. However, since TriMV is almost always found with WSMV [43], we were unable to obtain TriMV infected wheat tissue that was not also infected with WSMV in the necessary time frame; therefore, TriMV was not included in the specificity study. However, the primers used in this assay were confirmed to be WSMV specific through *in silico* analyses. Our *in vitro* specificity testing demonstrated that the genetically similar viruses and ecologically similar organisms included in this study would neither interfere with the assay nor provide false positives. This conclusion was confirmed by the lack of fluorescent signal during capillary electrophoresis of the SNaPshot products for HPV, CYDV-RPV and BYDV-PAV.

Implementation of this assay in a forensic investigation requires that the SNP profile of a single sample be reproducible, and this requirement was fulfilled through qualitative comparisons in this study. The inability to apply this assay in a quantitative capacity does not hamper its utility in forensic investigations, as the presence/absence of SNPs in a profile should provide sufficient information. However, quantitative analyses may be beneficial for epidemiological or ecological studies in which the amount of genetic change may provide insight into organismal interactions or disease development.

Even though a wheat virus was used as the model for the development of this assay, SNP typing may prove to be a successful molecular comparison tool for other organisms as well. Ultimately, the strength and reliability of the assay relies on appropriate marker selection which is facilitated by understanding the biology of the organism, the pressures that result in genetic evolution, and the functionally important regions of the genome. SNP analysis may provide additional benefits that other genetic analysis techniques do not. Specifically, DNA does not need to be intact to perform SNP analysis, which would be beneficial for analysis of fungi where the large genomic DNA may be easily sheared during processing.

This study demonstrates a new method for viral isolate comparison that could potentially be utilized in a forensic investigation. Before this method can be incorporated into the forensic science toolbox, a more thorough and exhaustive validation procedure must be conducted. Due to resource constraints, we were unable to perform this level of validation, which would include larger inclusivity and exclusivity panels and standardization of procedures to understand fully the variant representation and error rates. However, this preliminary evaluation of the technology supports the utility of this assay in a forensic setting and suggests a successful validation could be achieved.

## Conclusions

This specific, sensitive, and discriminatory SNaPshot assay has been developed using WSMV as a model system. Similar assays could be developed for application to many other plant, animal, or human pathogens by designing organism-specific primers, a task that is becoming ever more feasible with the increasing availability of microbial sequence data and primer designing software. This method already has been shown to be useful for other pathogens, such as *Potato virus* Y [15], and for genetic comparisons of humans and other organisms [7-9,11-13,44-47]. The assay uses molecular biology techniques that are well established already in many research and diagnostic laboratories, allowing for a seamless introduction of the technology into existing laboratories.

## Methods

### Selection of virus strains

WSMV strains were received from virus collections at Oklahoma State University in Stillwater, OK, the University of Nebraska in Lincoln, NE, and from AGWEST Plant Laboratories in South Perth, West Australia. Other infected wheat samples were collected in Oklahoma wheat fields and from the Great Plains Diagnostic Network (GPDN) wheat virus survey of 2008. Strains of ONMV*,* WSSMV*,* and MDMV strain A were received from a virus collection at Oklahoma State University. Strains of HPV*,* BYDV-PAV*,* and CYDV-RPV were received from the GPDN wheat virus survey. All samples were maintained as dry, frozen leaf material at −80°C, with the exception of the Australian WSMV isolate Ginnindera, which was stored at −80°C as an RNA pellet.

### SNP identification

SNPs were identified by comparing the sequences of 85 different WSMV sequences obtained from the NCBI database, including the strains Sidney 81 [GenBank: AF057533], Type [GenBank: AF285169.1], and El Batan 3 [GenBank: AF285170.1]. The CP and HC-Pro segments of the genome were targeted for the SNP-typing method because of the high variability among strains [17,18]. The polymorphic sites selected contained high variability among the strains and were surrounded by semi-conserved regions that would provide a suitable template for primer binding.

### Nucleic acid extraction

Total RNA was extracted from healthy or infected wheat (*Triticum aestivum)* leaves using the RNeasy Plant Mini Kit (Qiagen, Germantown, MD, USA) according to the manufacturer’s instructions. A total of 100 mg of tissue was homogenized at full speed (3,450 oscillations/minute) for 90 seconds using a mini bead-beater (Biospec Products, Bartlesville, OK, USA) and 550 μl of the RLT lysis buffer. The RNA was eluted using 50 μl of RNase-free water supplied with the kit and the concentration determined using a Nanodrop v.2000 spectrophotometer (Thermo Scientific, Waltham, MA, USA).

### Reaction controls

A synthetic plasmid carrying the target gene sequences derived from the cloning vector pUC57 was created and produced (GenScript, Piscataway, NJ, USA) for each of the target regions of WSMV to serve as a positive control. The CP synthetic plasmid contains nt 8061–9428 (1,367 bp) and the HC-Pro synthetic plasmid contains nt 1081–2507 (1,426 bp) (based on Sidney 81 genome [GenBank: AF057533]). The plasmids were used during the one-step RT-PCR at a concentration of 2 ng/μl and were processed in the same fashion as the samples.

In addition to the positive control, a negative control was included with each sample set in every process. The negative control consisted of all reaction reagents with sterile nanopure water instead of the viral sample. These controls were used to verify the performance of the assay and confirm the lack of contamination. Additionally, Sidney 81 derived synthetic plasmids confirm the generated profile remains authentic after the sample is subjected to PCR.

### cDNA synthesis and amplification

cDNA was synthesized and amplified from total RNA in a single step using the SuperScript™ III One-Step RT-PCR kit with Platinum© *Taq* (Invitrogen, Carlsbad, CA, USA)*,* following the protocol supplied by the manufacturer. Briefly, in the first part of the RT-PCR, primer C1 (5′-TACTTGACTGGGACCCGAA-3′; Sidney 81 nt 8117 to 8134) was used for the CP reverse-transcription and primer HCR (5′- CATGCTTGTATACTGAGAACAGTCTCTTG-3′; Sidney 81 nt 2373 to 2345) was used for the HC-Pro reverse-transcription, in separate reactions, to synthesize the DNA strand complementary to the RNA sequence [16,48,49]. In the second part, the CP region of the cDNA was amplified using the forward primer C1 and the reverse primer XC1 (5′-AACCCACACATAGCTACCAAG-3′; Sidney 81 nt 9371 to 9351) [16,29,48,49]. The HC-Pro region of the cDNA was amplified using the forward primer HCF (5′- GAAATGCACACATGGACTTAGATGGTAT-3′; Sidney 81 nt 1159 to 1186) and the reverse primer HCR [50]. These primers produced approximately 1.3 kb amplicons for both CP and HC-Pro which contained the gene and flanking regions (based on the Sidney 81 genome [GenBank: AF057533]).

Each RT-PCR reaction contained the following components: 25 μl 2X reaction mix (supplied with kit), 1 μg template RNA (6 ng of synthetic plasmid), 0.2 μM of each primer (C1 and XC1, or HCF and HCR), 2 mM MgSO_4_, 1 μl of RT/Platinum *Taq* mix (supplied with kit), and sterile nanopure water (17 Ω) up to 50 μl. The kit protocol consisted of cDNA synthesis (one cycle of 50°C for 60 minutes and 94°C for two minutes), amplification (35 cycles of 94°C for 30 seconds, 55°C for 30 seconds, and 68°C for one minute) and elongation (68°C for five minutes).

The amplification of the CP and HC-Pro genes was verified by electrophoresis using a 1.5% agarose gel in 1X TAE buffer containing SYBR® Safe DNA gel stain (Invitrogen). The samples and a 100 bp DNA ladder (Invitrogen) were loaded with 1X loading dye containing bromophenol blue and xylene cyanol and were electrophoresed for 30 minutes at 100 mV. The gel was visualized with a UV-transilluminator and photographed using a camera. The RT-PCR amplification products were treated with a mixture containing one unit of shrimp alkaline phosphatase (SAP), two units of exonuclease 1 (Exo1) (Affymetrix, Inc., Cleveland, OH, USA), and sterile water up to 16 μl for 30 minutes at 37°C followed by 75°C for 15 minutes to inactivate the enzymes. Samples were used immediately in SNaPshot assays or stored at −20°C.

### SNaPshot technology

The PRISM® SNaPshot™ Multiplex Kit (Applied Biosystems, Foster City, CA, USA) was used to generate genetic fingerprints from each PCR product. The methods below briefly describe the manufacturer’s process.

Specific WSMV primers were designed for each targeted SNP (Table 1). Each primer was of a different length, ranging from 30 to 65 nt, to facilitate product recognition. The primers were complementary to the DNA template beginning one nucleotide 3′ of the polymorphic site. Controls that were supplied with the SNaPshot kit were included in each assay to verify the performance of the PCR components.

Each reaction contained 5 μl SNaPshot Multiplex Ready Reaction Mix (provided with kit), 3 μl purified PCR product, 1 μl of pooled SNaPshot primers (final concentration of each ranging from 0.1 to 0.4 μM each; see Table 1), and 1 μl of sterile nanopure water (17 Ω). The products were amplified using the following two-step program: 25 cycles of 96°C for 10 seconds and 60°C for 35 seconds. The amplified products were treated with SAP to prevent further binding of ddNTPs. One μl (1 unit) of SAP, 2 μl of 10X SAP buffer, and 7 μl of nuclease-free water were added to each SNaPshot reaction. The mixtures were incubated for 30 minutes at 37°C followed by 15 minutes incubation at 75°C.

The SNaPshot products were separated by capillary electrophoresis on an ABI 3730 Genetic Analyzer (Applied Biosystems) after combining 0.5 μl purified SNaPshot product, 0.5 μl GeneScan 120-LIZ size standard, and 9 μl Hi-Di Formamide (Applied Biosystems). The parameters suggested in the SNaPshot protocol were used. The separated products were analyzed using Peak Scanner Software v1.1 (Applied Biosystems).

### Data analysis

Multiple datasets were created from the SNP profiles for quantitative analyses, including data from each peak having a peak height greater than 800 FUs (this limit was set based on background noise, explained in results section). Of the first two datasets created using peak height values, one was composed of the absolute fluorescence values after normalization based on positive control values; the second was composed of percentages of each nucleotide at each SNP position. Two additional datasets were created similarly, but based on the area under the curve instead of peak height. Both peak height and area under the curve were included in the analyses to determine which set of fluorescence values was more reliable for profile comparisons, determined through statistical analyses. Each dataset was analyzed using ANOVA, assuming a randomized complete block model and a *P*-value <0.05. The strain and the SNP position were the random blocking effects and the replicate was the factor of interest to assess reproducibility.

Using the percent nucleotide dataset from the area under the fluorescent peak values, a PCA was performed using Canoco software (Biometris-Plant Research International, Wageningen, The Netherlands) with square-root transformation [51]. The WSMV strains and field isolates were included in the analysis, in addition to the *in silico* SNP profile data for strains El Batan 3 and Czech to provide a known dimension of diversity.

## **Abbreviations**

ABI: Applied Biosystems Inc.; ANOVA: Analysis of variance; BLASTn: Basic local alignment search tool for nucleotides; bp: Base pair; BYDV-PAV: Barley yellow dwarf virus PAV; CP: Coat protein; CYDV-RPV: Cereal yellow dwarf virus RPV; ddNTPs: Dideoxynucleoside triphosphates; Exo1: Exonuclease 1; FUs: Fluorescent units; GPDN: Great Plains Diagnostic Network; HC-Pro: Helper component – protease; HPV: High plains virus; MDMV: Maize dwarf mosaic virus; NCBI: National Center for Biotechnology Information; nt: Nucleotide; ONMV: Oat necrotic mottle virus; P1: Protein 1; P3: Protein 3; PCA: Principal component analysis; PCR: Polymerase chain reaction; RT-PCR: Reverse transcription polymerase chain reaction; SAP: Shrimp alkaline phosphatase; SNP: Single nucleotide polymorphism; STR: Short tandem repeat; TAE: Tris-acetate-EDTA; TriMV: Triticum mosaic virus; US: United States; UV: Ultra-violet; WSM: Wheat streak mosaic; WSMV: Wheat streak mosaic virus; WSSMV: Wheat spindle streak mosaic virus.

## Competing interests

The authors declare they have no competing interests.

## Authors’ contributions

SMR participated in the design of the study and carried out the experimental design, genotyping, data analysis, and drafted the manuscript. MP participated in the design of the study and performed the statistical analysis. RWA conceived of the study and participated in the design of study and data analysis. UM participated in design of the study and data analysis. JC participated in the concept and design of the study and genotyping. JF participated in conception and design of study, data analysis, and drafting of manuscript. All authors read and reviewed the final manuscript.
